# The Institutional Library

**Published:** 1916-11-11

**Authors:** 


### THE INSTITUTIONAL LIBRARY.

### SCHOOL MEDICAL INSPECTION.

**The Nation of the Future.** By L. HADEN GUEST, M.R.C.S., L.R.C.P. Illustrated with eight photographs. (London: G. Bell and Sons. 1916. Price 2s. net.)

THE author of this little work writes from a camp in France, where the men are equipped with all they need for hard fighting, but he rightly says that "they cannot be equipped with a physical health and efficiency greater than their childhood has left them. Only care of childhood can give us adult men of that force and vigour which is latent in our race, but which often bad conditions deform or suppress." As school medical officer to the London County Council, he not only describes the routine of medical inspection and the methods adopted by the medical examiner, but also points out in forcible language the pressing need for such physical supervision of the children. Dr. Guest shows that of all abnormal physical conditions that of malnutrition is the most important. A suggestion of 10 per cent. is made for the proportion of the "poverty group" among the school population. It is from this group that parasitic reinfection constantly occurs, and among it may be found the greater number of the cases of malnutrition, while from it epidemics of infectious disease preponderantly spring. Bearing in mind the manner in which a child in this group is handicapped by bad home surroundings, indifference or neglect of parents, a good case is made out for the school clinic, which the author would like to see established in every great centre. Altogether a thoughtful and instructive little book.

**Newsholme's School Hygiene. The Laws of Health in Relation to School Life.** New Edition, rewritten by JAMES KERR, M.A., M.D. (London: G. Allen and Unwin. Crown 8vo, pp. 348.)

THIS popular text-book, of which 22,000 copies had already been published in thirteen editions, has now been entirely rewritten by Dr. James Kerr. A favourite so long and completely established scarcely needs commendation to the public to which it appeals; but as the book has been completely rewritten by a new hand, it requires more notice than an ordinary new edition. Dr. Kerr has done his work so well as to leave little room for criticism. The first twenty pages are occupied with "Physiology, Psychology, and Ethics." There is, however, scarcely any reference to the last, an omission that is not to be regretted, and the other subjects also might as well have been omitted. Dr. Kerr's description is as good as could be given in the space he allows himself, but to suppose that anyone to whom the subject is new can pick up a knowledge of it from twenty pages of description is chimerical. The chapter on "Age and Sex" is full of good sense, and the warning that children under the age of six or seven should never be allowed to do near eye-work or finger-work, though it is stated too absolutely, is wholesome. In another matter the warning might have been more emphatic. "When a child is growing rapidly in height, probably his powers of mental application greatly diminish." This is true both of growth in height and of growth in bulk. Children grow not uniformly, but by fits and starts, and the time of rapid growth is a time of danger, which is greater the more rapid the growth. When they are growing rapidly, lessons should be eased off, and when they are growing very rapidly, lessons should be stopped altogether. Many a child has broken down utterly from his education being forced while he has been in very rapid growth, especially between the ages of fourteen and sixteen. It is doubtful whether much information is conveyed by the assertion that "fatigue is chiefly subjective." If it means that fatigue is known chiefly by the feeling of the fatigued person, it would be better to say so. The terms "subjective" and "objective" may mean anything, and very often mean nothing. The chapters on defective children are excellent, but the section on punishment is meagre. In a future edition, which is sure to be called for before long, Dr. Kerr might elaborate this a little, and insist upon the punishment being made to fit the crime. He speaks as if punishment necessarily meant corporal punishment. In correcting faults, nothing is more important than that punishment should be appropriate, and in nothing can the ingenuity of the schoolmaster be more usefully employed than in devising appropriate punishments—that is to say, punishments that shall make the child feel and understand the wrongness of his fault. Dr. Kerr's book, like its predecessor, will no doubt become a classic, and should be in the hands of every schoolmaster.

**A Practical Guide to X-Rays, Electro-Therapeutics and Radium Therapy.** By Major A. E. WALTER, I.M.S. (Calcutta and Simla: Thacker, Spink and Co. 10s. 6d. net.)

THIS excellent little treatise may be regarded in some respects as a second edition of Major Walter's "X-rays in General Practice," published in 1906, though the work has been re-written in the light of later experience, and includes in addition electro-therapeutics generally as well as radium therapy. The author draws attention to the difficulties arising from the conditions of work and climatic influences in India, and illustrates methods based on his experience for surmounting these difficulties. He refers to the improvement in the technique of radiography since the adoption of the couch, and to the fact that the type of couch supplied with the three-ply wooden top will not withstand a tropical climate. One of the outstanding features of the work is the exceptionally lucid and practical introduction to elementary electricity, ignorance of which is the stumbling-block of many students and practitioners of radiography and electro-therapeutics. Nor has the author overlooked the need for a knowledge of the mechanical and electro-mechanical devices for producing electric current in the absence of a general supply. The chapter on photography might with advantage have been fuller. A knowledge of photography in *x*-ray work, in our opinion, stands next in importance to a knowledge of elementary electricity; it is not an uncommon observation that the best technicians in radiography are those who have an expert knowledge of photography. The use of *x*-rays in dental surgery is referred to, and the value of the intra-oral film; there is ample scope for a wider application of radiography to this branch of surgery. We do not agree that the method referred to is the only satisfactory one; a very useful extra-oral plate may be obtained of each side of the jaw separately by focussing over the mastoid process. The chapters on electro-therapeutics and radium therapy are lucidly written, and should offer an excellent groundwork for wider study. Although the reproductions of some of the diagnostic plates do not, possibly, do justice to the originals, the book is nevertheless nicely produced, and the method of arrangement and lucid style should obtain for it a popular position among the standard text-books.

### THE DIAGNOSIS AND TREATMENT OF PHTHISIS.

**Pulmonary Tuberculosis in General Practice.** By Halliday G. Sutherland, M.D. (London: Cassell and Co. 1916. Pp. 290. Price 10s. 6d. net.)

The author of this text-book, in common with many other tuberculosis officers, has had abundant opportunities of witnessing the results of what he aptly terms "the tragedies of general practice" in relation to the early diagnosis of pulmonary consumption. He rightly bewails the hesitancy in approaching the problem and the months of "ill-timed dalliance waiting for signs of advanced disease." The busy practitioner, rather than condemn his patient too hastily to sanatorium exile, is sorely tempted to postpone the fatal pronouncement until the appearance of some definite symptom or physical sign renders the diagnosis irrefutable to everybody. Too often the early manifestations of phthisis are totally ignored, or are mistaken for indications of some other malady. In this popular monograph Dr. Sutherland has conspicuously succeeded in giving the practitioner just what he wants—a concise handbook, embodying the solution of all the points that constantly present themselves in connection with cases of pulmonary consumption. Tuberculin treatment is dealt with in a somewhat restrained fashion. It is rightly acknowledged to be a double-edged weapon, but "is harmless if used with judgment, knowledge, and care." It is not claimed as a specific cure, but as a valuable adjunct to general treatment. The importance of a routine laryngoscopic examination is insisted upon.

In eighteen chapters and two appendices every phase of pulmonary consumption is dealt with, and the sections on treatment are particularly full. It may be noted that the author does not favour the administration of nascent iodine, nor is he enthusiastic as regards the use of antiseptic inhalations. He has, however, found the daily inunction of guaiacol into the skin of the thorax "of considerable value." The last chapter is occupied with a description of the method of producing artificial pneumothorax and surgical treatment, which methods "have a limited application in selected cases." The practitioner's library is incomplete if this volume has no place therein.

**Bedsores: Their Prevention and Cure.** By C. W. Smart. (London: J. Bale, Son and Danielsson. 1s. net.)

This book is written in an amateurish and discursive style. There is a great deal of valuable information in it, which might be of help to amateur nurses in home nursing, but it is not a book which will be of much use to the probationer or trained nurse.

**Clinical Notes for Probationers.** By Felicie Norton. (London: The Scientific Press, Ltd. 1s. net.)

This little book contains a great deal of very valuable information which cannot fail to be of use to the certificated nurse as well as to the probationer. The information is put clearly and concisely, and has the merit of being very necessary for a nurse to know, though it is not always forthcoming in many so-called nursing books. An index would have been an advantage, as well as notes on the preparation of instruments, gloves, and hands for dressings, and a list of the usual necessaries for an ordinary dressing in the ward. We miss, too, any reference to the making-up of lotions, their usual strength for stock purposes, and an explanation of the principal use of each. Many probationers find the figures very bewildering. Perhaps Miss Norton may be able to add notes on these subjects to a subsequent edition.

**The Rhymes of a Red Cross Man.** By R. W. Service (London: Fisher Unwin. 1916. Pp. 176. Price 3s. 6d. net.)

From the reputation which Mr. Service has long had as an interpreter of the Yukon, and of the hardships and heroisms of those who live there, it is obvious that he possesses the power of seeing and of making others see both the broad outlines and also the details of life in primitive conditions. It is therefore not to be wondered at that the life of the soldiery in France—and their deaths not less than their lives—should move him to descriptive verses. Those of delicate susceptibilities had better avoid Mr. Service's Red Cross rhymes; for he is not the man to mince words when dealing with horrible things. But for those who can face strong meat and want to get an idea of the ghastly side of war Mr. Service is a guide whom they can trust for realism relieved by occasional humour. Poetry there is none in this volume, but then the author himself nowhere seems to claim that there is. He might, however, have restrained his publisher from ascribing these rhymes to the "Canadian Kipling," an injudicious overstatement which is doubtless as unfair to Canada as it certainly is to Mr. Kipling.

**Medical Reporting in Pitman's Shortland.** By H. Dickinson. (London: Pitman. 3s. net.)

This work, by perhaps the best-known exponent of the art of shorthand reporting as applied to the domain of the physician and surgeon, will be found of great value to the medical man's amanuensis, and to reporters of medical lectures and discussions. It may be commended, too, to the attention of all those who are engaged, whether as reporters or as shorthand amanuenses, in highly technical work of any kind, as showing the necessity of supplementing the ordinary shorthand text-books by the careful study of the special phraseology employed in the particular department in question. The volume contains an exhaustive list of medical phrases with their shorthand outlines, as well as an interesting preface and a number of exercises culled from the author's own notebooks, being actual specimens of medical lectures, etc., reported by himself in the course of his professional career. We congratulate Mr. Dickinson on the production of a book so well calculated to assist the conscientious shorthand-writer in one of the most difficult branches of his art.

**Low's Handbook to the Charities of London, 1916.** (London: Sampson Low, Marston and Co., Ltd. 1916. Price 1s. net.)

The institutions in this handbook are entered alphabetically under their formal titles, but a classified table of contents facilitates reference to any group of charities. A small proportion of the titles of the institutions listed in the main body of the book are printed in capitals, the remainder in more modest type. The differentiation apparently has nothing to do with the relative importance of the charities, the reason being that the former class make use of the advertisement pages. The information given as to receipts and expenditure is not claimed to apply to any given year. A preface deals exclusively with the description of the new King's College Hospital at Denmark Hill.

